# Optical phase retrieving of a projected object by employing a differentiation of a single pattern of two-beam interference

**DOI:** 10.1038/s41598-023-41627-y

**Published:** 2023-09-08

**Authors:** W. A. Ramadan, A. S. El-Tawargy, H. H. Wahba

**Affiliations:** 1 Physics Department, Faculty of Science, Damietta University, New Damietta City, 34517 Egypt; 2 Physics Department, Faculty of Science, Taif University, 21974 Taif, Al-Haweiah Saudi Arabia

**Keywords:** Optics and photonics, Physics

## Abstract

In this work, we present a new approach to retrieve the optical phase map of an object which is projected by a single differentiated two-beam interference pattern. This approach is based on the differentiation of the intensity equation of the two-beam interference with respect to the carrier’s phase angle. Therefore, two interference patterns which are shifted by a very small phase angle can be obtained. Then, these two patterns are projected on the object. By exploiting the definition of the mathematical differentiation, the optical phase object’s variations are retrieved from the recorded intensity distributions of both projected patterns. According to this method, the extracted optical phase angles are raised as an inverse “*sin*” function. This means that the unwrapping process of this function limits the recovered phase angles between − *π*/2 and *π*/2. So, the unwrapping process of these unusual wrapped phase angles is explained. The proposed method is applied on (a) two objects which are simulated by combinations of multiple Gaussian functions and (b) a 3D real object. It is found that the inclination of the projected interference pattern on the object redistributes the intensity distribution due to the Lamber’s “*cos*” aw of illumination. This effect is considered in the retrieving process of the object’s phase map. The limitations of the presented method are discussed and the obtained results are found promising.

## Introduction

The three-dimensional (3D) real-time measurements of surface shapes are extremely important in different fields such as optics, engineering, industry, medicine, etc.…^1,2^. Optically-based profilometry techniques are commonly utilized to obtain 3D real-time surface shaping since they are considered noncontact and nondestructive tools^1,3–5^. Therefore, one can find different attempts which combine projection of interference fringes and phase-shifting methods for this purpose^1,2,6–9^. This combination is preferable for two reasons. On one hand, fringe-projection is based on the projection of pre-defined interference fringes on an object in order to change their phase distribution due to the shape of this object’s tomography^1,2,5–8^. On the other hand, the phase-shifting methods can be used for “pixel by pixel” extraction of an interference pattern’s phase distribution with high resolutions^1,2,5,10–12^.

By definition, the phase-shifting process requires more than one interference pattern, usually three or four^2,5,9–11^ (or even more^13^) phase-shifted interference patterns with known phase shifts. These shifted patterns can be used to extract the optical phase information with high accuracy. But, retrieving the phase values from multiple images is a time-consuming process which persecutes the real-time measurements^1,2^. Therefore, recent attempts are presented to overcome this problem; e.g., algorithms based on only two-step phase-shifting of a certain phase shift which is capable of speeding up the phase retrieving process with keeping the same quality level of the retrieved object’s shape^1,2,12^. Despite considering more than one pattern, the phase-shifting profilometry is still preferable compared to Fourier-transform profilometry which requires only one image for phase extraction. This is due to the influence of noise, filtration window and the boundary defects on the results’ accuracy in case of the Fourier transform profilometry^2^.

For the two-step phase-shifting methods, there are only two equations which can be established. These two equations are less than the three unknown parameters; (1) background term (or carrier), (2) intensity and (3) phase of each pixel in the interference pattern^1,2,7,14,15^. Therefore, different approaches are presented to extract the phase data using the two-step phase-shifting methods such as those based on the slow variable background term and the specific phase-shift^2,12,16,17^. Moreover, it was reported that the intensity of the slow variable background of the fringe patterns (i.e. carrier fringes), shifted by π/2^1^, can be removed using an intensity derivative approach. The differentiation, in that approach, was respect to the position in the *x*-direction. The differentiation was performed on the two images (containing the object) and having a difference in phase equals *π*/2. We think that the differentiation in this case leads to transferring the position of the object when it is imaged. Additionally, that approach is only applicable when there is a variation of phase in the *y*-direction while the variation in *x*-direction could not be evaluated correctly.

Recently, an approach to retrieve the 3D surface map of an estimated object by projection of a couple of two-beam interference patterns was presented^5^. In that approach, a couple of two-beam interference patterns were produced to be shifted by a phase angle (π/2) and differentiated with respect to the carrier’s phase angle. For retrieving the phase object, four interference patterns were needed to be projected on the objects. Due to the (π/2) shift between the patterns, the resultant phase map is extracted by employing the conventional unwrapping process, i.e., inverse “*tan*” unwrapping. No real object was used in that work.

In the presented work, a developed approach is presented where a single two-beam intensity distribution is utilized. The two-beam intensity distribution is differentiated with respect to the carrier’s phase angle before projecting on the phase object. Therefore, the projected patterns are only two patterns with a small phase-shifting angle in between. Two estimated 3D objects are used to retrieve their phase maps in order to evaluate the accuracy of the method. Additionally, the method is applied on a 3D real object for demonstration of its validity.

## Theory

The simplest form of the 1D intensity distribution of two-beam interference (say, in *x*-direction) can be given as follows:

1 $${I}_{c}(x)={I}_{1 }+{ I}_{2 }+2 \sqrt{{I}_{1\mathit{ }}{I}_{2\mathit{ }}} {\text{cos}} \left(\delta \left(x\right)\right),$$

where, *I*_*c*_(*x*) can be called the carrier’s intensity distribution in the *x-*direction while the constants *I*_*1*_ and* I*_*2*_ are the intensities of the first and second interfered beams, respectively. *δ*(*x*) is the 1D spatial optical phase difference between the phases of the two interfered beams and it can be denoted as the phase angle of the carrier’s frequency.

The values of* δ*(*x*) can be reconstructed by, firstly, differentiation of Eq. (1) with respect to* δ*(*x*) which gives:

2 $$\frac{\partial {I}_{c}(x)}{\partial \delta (x)}= - 2 \sqrt{{I}_{1}{ I}_{2}}{\text{sin}}\left(\delta \left(x\right)\right),$$

then, from the definition of the mathematical differentiation, we can write:

3 $$\frac{\partial {I}_{c}(x)}{\partial \delta \left(x\right)}= \underset{\epsilon \to 0}{\mathit{lim}}\left(\frac{{I}_{c,\epsilon }\left(x\right)-{I}_{c}(x)}{\epsilon }\right)= - 2 \sqrt{{I}_{1}{ I}_{2}}{\text{sin}}(\delta \left(x\right)),$$

where, *I*_*c,ϵ*_(*x*) is the carrier’s intensity when *δ*(*x*) suffers an infinitesimal variation (*ϵ*), i.e.:

4 $${I}_{c,\epsilon }\left(x\right)={I}_{1 }+{ I}_{2 }+2 \sqrt{{I}_{1\mathit{ }}{I}_{2\mathit{ }}}\text{ cos} \left(\delta \left(x\right)+\epsilon \right).$$

Therefore, Eq. (3) can be rewritten as follows:

5 $$\delta \left(x\right)={\mathrm{sin}}^{-1}\left(\frac{- {(I}_{c,\epsilon }\left(x\right)-{I}_{c}(\mathrm{x}))}{ 2 \epsilon \sqrt{{I}_{1 }{I}_{2 }}}\right).$$

Equation (5) provides the phase angles distribution in the terms of inverse “*sin*”. So, the recovered wrapped phase angles will be limited between − π/2 and π/2 which is not usual to be obtained^18–20^. The unwrapping process in this case, along the *x*-direction, can be performed by dividing the phase angles into small intervals where each one of them represents the phase at ${\delta }_{j+1}\left(x\right)-{\delta }_{j}\left(x\right)$ along the *x-*direction. Then, running the summation over these intervals from *j* = 1 to* j* = *M*−1, where* M* is the total number of pixels in the *x*-direction. Therefore, the unwrapped phase angles of the carrier pattern *Δ*_*c*_(*x*) can be obtained using the following formula.

6 $${\Delta }_{c}\left(x\right)=\sum_{j=1}^{M-1}\left|{\delta }_{j+1}\left(x\right)-{\delta }_{j}\left(x\right)\right|,$$

where, *j* is the pixel’s order along the *x-*direction.

The above simple mathematical treatment means that the values of *δ*(*x*) can be retrieved using Eq. (5) for the two intensity distributions described by Eqs. (1) and (4) which represent 1D phase variations. The 2D interference patterns can be obtained by repeating these 1D phase variations in the *y*-direction. These two resultant patterns represent a pair of carrier fringes which are separated by the phase shift (*ϵ*). The value of *ϵ* is supposed to equal *2πf* /*M*, where *f* is the spatial frequency of the fringes distributed over the image’s width *M* (pixels), i.e., *ϵ* is equivalent to only one pixel. The phase shift (*ϵ*) is too small to be observed by the naked eye.

In the presented study, we prepared three pairs of interference patterns with three different values (*f* = 0.5, 3 and 12). For more details about these interference patterns, see Supplementary Appendix 1. The purpose of these different frequencies will be clarified later. Each pair of these images has its own value of *ϵ* where *M* = 2000 pixels is kept fixed for all patterns. If each pair of these patterns is projected on a plane containing an object, they almost provide the same light intensities at each corresponding point on the object plane. This is due to the small value of *ϵ.* Therefore, many problems related to significant change in the intensities illuminating the object, e.g. reflectivity, color and the projector nonlinear response to the computer-generated signal could be avoided^1^.

Now, assume that the projected plane contains a 3D object which can cause a change in the optical phase’s distribution by the function *φ* (*x*,*y*). In this case, the intensity distribution in the presence of the object can be written as follows:

7 $${I}_{o}(x,y)={I}_{1 }+{ I}_{2 }+2 \sqrt{{I}_{1\mathit{ }} {I}_{2\mathit{ }}} {\text{cos}}\left(\delta \left(x\right)+\varphi (x,y)\right).$$

Similarly, when the phase angles *δ*(*x*) is changed with the small value *ϵ,* the intensity distribution takes the following form:

8 $${I}_{o,\epsilon }\left(x,y\right)={I}_{1 }+{ I}_{2 }+2 \sqrt{{I}_{1 } {I}_{2 }}\mathrm{ cos}\left(\delta \left(x\right)+\varphi \left(x,y\right)+\epsilon \right).$$

Following the same procedure described above (providing that $\varphi \left(x,y\right)$ is not differentiable with respect to *δ*(*x*) because it depends on the rigid shape of the object), we can obtain the modified carrier phase angles distribution which is affected by the phase object as follows:

9 $$\delta \left(x\right)+\varphi (x,y)={\text{sin}}^{-1}\left(\frac{{- (I}_{o,\epsilon }\left(x,y\right)-{I}_{o}(x,y))}{ 2 \epsilon \sqrt{{I}_{1 } {I}_{2 }}}\right).$$

Similarly, the unwrapping process, described above, can be performed by applying the following summation where the same limitation of the inverse “*sin*” function is, also, raised here.

10 $${\Delta }_{c,o}(x,y)=\sum_{j=1}^{ M-1}\left|({\delta }_{j+1}\left(x\right)+{\varphi }_{j+1}(x,y))-({\delta }_{j}\left(x\right)+{\varphi }_{j}\left(x,y\right))\right|.$$

The extraction of the object optical phase map *φ* (*x*,*y*) can be obtained by subtracting Eq. (6) from Eq. (10).

11 $${\varphi \left(x,y\right)= \Delta }_{c,o}\left(x,y\right)- {\Delta }_{c}\left(x\right).$$

## Estimation of 3D objects

In this stage, we prefer to apply this approach on two estimated objects in order to make a confident evaluation of the proposed method before applying on a real object. Each of these estimated objects consists of a combination of nine 3D Gaussian formulae as follows:

12 $${{G}_{T}\left(\mathrm{x},\mathrm{y}\right)= \sum_{n=1}^{n=9}G}_{n}\left(x,y\right),$$

where, ${\mathrm{G}}_{n}(x,y)$ is given as:

13 $${\mathrm{G}}_{n}(x,y)={A}_{n} {e}^{- \frac{1}{2\left(1-{\rho }_{n}^{2}\right)}\left[{\left(\frac{x-{a}_{n}}{{w}_{xn}}\right)}^{2}-2{\rho }_{n}\left(\frac{x-{a}_{n}}{{w}_{xn}}\right)\left(\frac{y-{b}_{n}}{{w}_{yn}}\right)+{\left(\frac{y-{b}_{n}}{{w}_{yn}}\right)}^{2}\right].}$$

**Figure 1:**
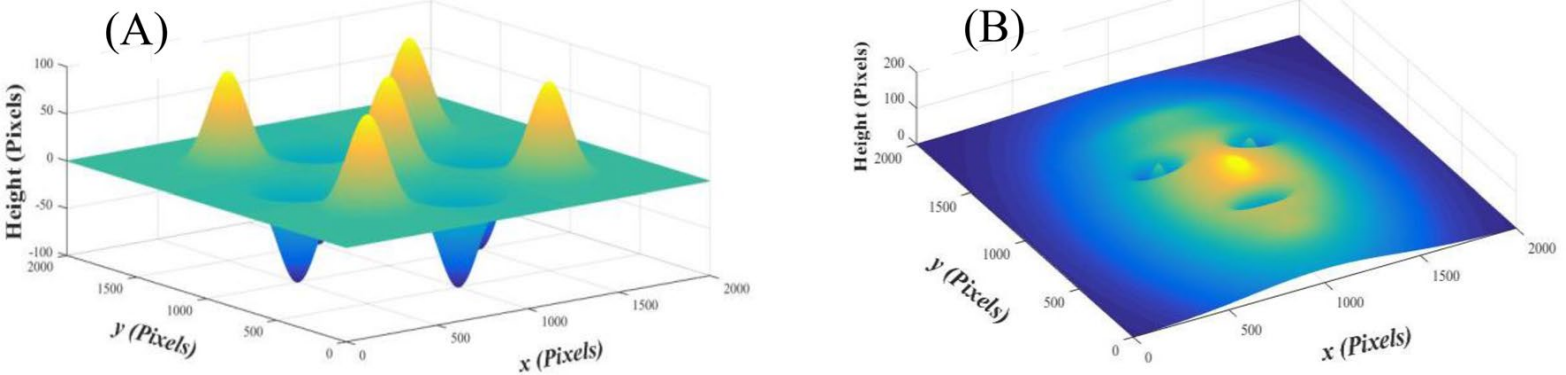
Two estimated objects consisting of nine 3D Gaussians forming the shapes of (**A**) eggs’ cardboard box and (**B**) mask-like face.

These 3D Gaussians are distributed on a plane of (2000 pixels × 2000 pixels) with different shape parameters, see Fig. 1. More information about the parameters of the surface objects can be found in Supplementary Appendix 2.

## Projecting an estimated object by a pair of two-beam patterns

### Change of phase due to the inclination of the incident light on the object

**Figure 2:**
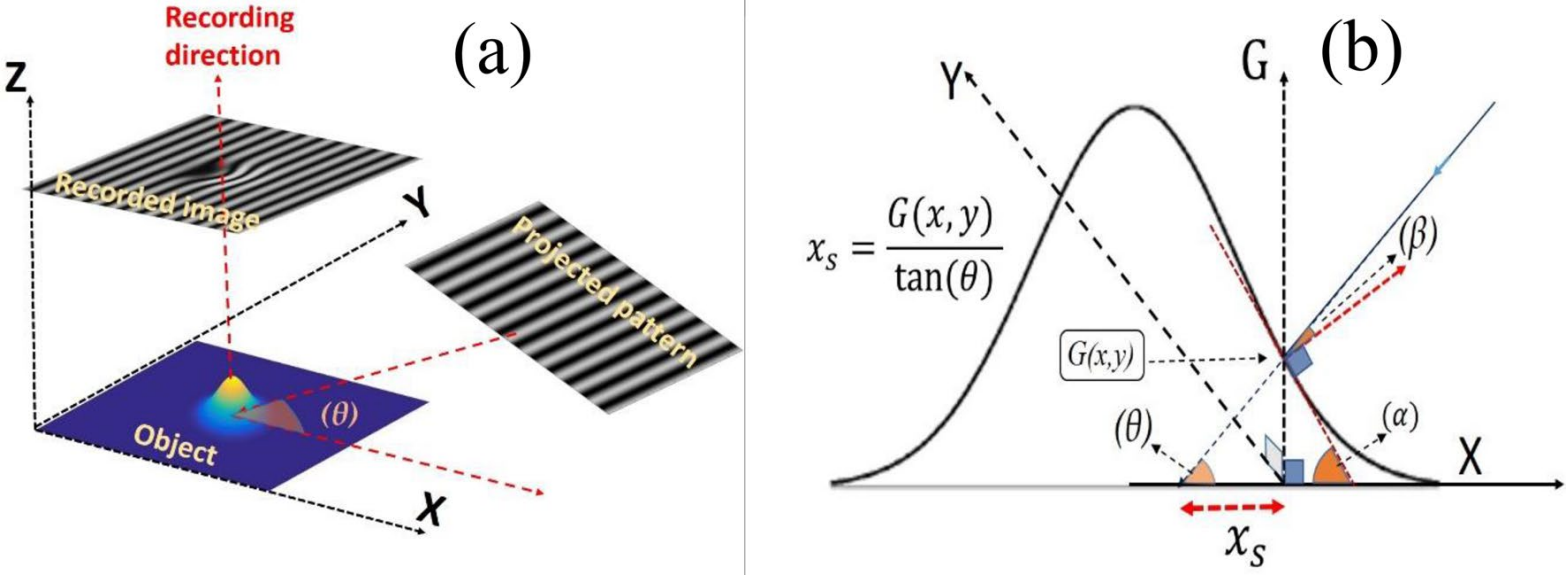
(**a**) The projected interference pattern on a phase object, with an inclination angle (*θ*), and the corresponding recorded image. (**b**) A geometrical representation of the relation between (*G*_*T*_(*x*,*y*)) and *x*_*s*_.

When a pair of two-beam interference images is projected on the object with an inclination angle (*θ*) between the light direction and the *x*-axis, the recorded fringes will be shifted according to the object’s height. This shift draws, in some way, the object’s topography. In this case, we consider the observer (recording camera) is facing the plane containing the object, see Fig. 2a. The relation between the object’s height (*G*_*T*_(*x*,*y*)) and the produced horizontal shift (*x*_*s*_) is depicted in Fig. 2b. So, the phase angles produced by the object are given as follows:

14 $$\varphi \left(x,y\right)=\xi \frac{{G}_{T}\left(x,y\right)}{{\text{tan}}\theta },$$

where, *ξ* (= 2*πf/M* (Rad./pixel)).

By substituting *φ* (*x*,*y*) in Eqs. (7) and (8), one can obtain the recorded estimated images of the two-beam interference which are deformed due to the presence of the object.

### Change of intensity due to the inclination of the incident light on the object

Up to this point, we have the equations describing the variation of the phase due to the presence of the object. In fact, this object does not only deform the phase distribution of the projected image but it, also, redistributes the projected intensities. This is due to the inclination angle (*β*) between the incident light and the object’s surface at each point, see Fig. 2b.

15 $$\beta \left(x,y\right)=\left(\theta \left(x,y\right)+\alpha \left(x,y\right)\right)-\frac{\pi }{2},$$

where, *α* is given by Eq. (16) and it can be either positive or negative.

16 $$\alpha =- {\mathrm{tan}}^{-1}\left(\frac{{\partial \mathrm{G}}_{T}\left(x,y\right)}{\partial x}\right).$$

This inclination raises a change in the fringes’ intensity distribution on the object according to the Lambert’s “cos” law of illumination^21^. In fact, the effect of Lambert’s “cos” law must be considered when dealing with the intensities *I*_1_ and *I*_2_. This means that *I*_1_ and *I*_2_ are assumed to be homogenous illuminations in case of illuminating a plane. But, when illuminating a plane containing an object, *I*_1_ and *I*_2_ have to be projected directly on the object. Then, their recorded images (which are not homogeneous anymore) should be used instead of the constants *I*_1_ and *I*_2_. However, in our case of estimated objects with known parameters and controlled incidence angle, we are able to expect this intensity distributions *I*_1_(*x*,*y*) and *I*_2_(*x*,*y*) as follows:

17 $${I}_{i}\left(x,y\right)={I}_{i}\mathrm{cos}(\beta (x,y)) i = 1 \mathrm{and} 2 .$$

**Figure 3:**
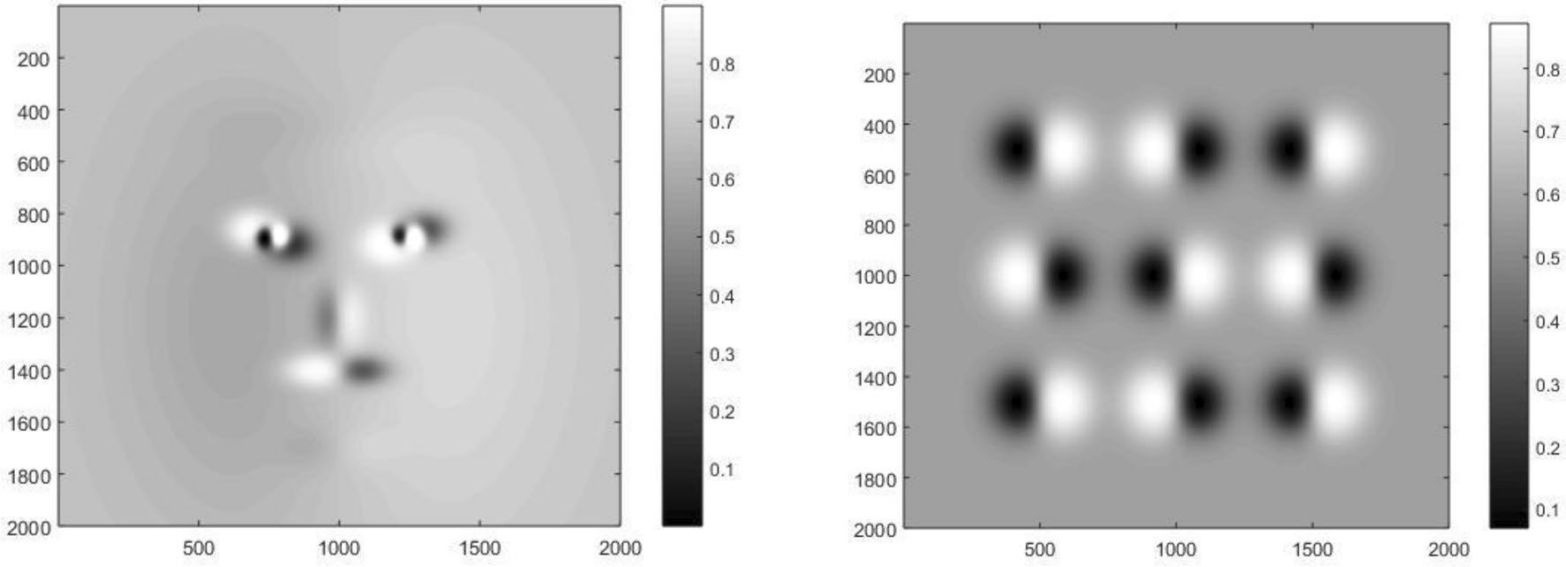
Illuminating the two estimated objects by the intensity distribution *I*_2_(*x*,y), given by Eq. (17).

Figure 3 illustrates how the two estimated objects seem when they are illuminated by the intensity distribution *I*_2_(*x*,y). It is clear that the illuminations are not homogeneous but they are rather redistributed due to the presence of the objects.

Regarding Eqs. (7) and (8), they can be rewritten, when the effect of the inclination angle is considered, as follows:

18 $${{I}_{O}^{L}=I}_{o}\left(x,y\right)\mathrm{cos}\left(\beta \left(x,y\right)\right),$$

and

19 $${{I}_{o,\epsilon }^{L}=I}_{o,\epsilon }\left(x,y\right)\mathrm{cos}(\beta (x,y)).$$

**Figure 4:**
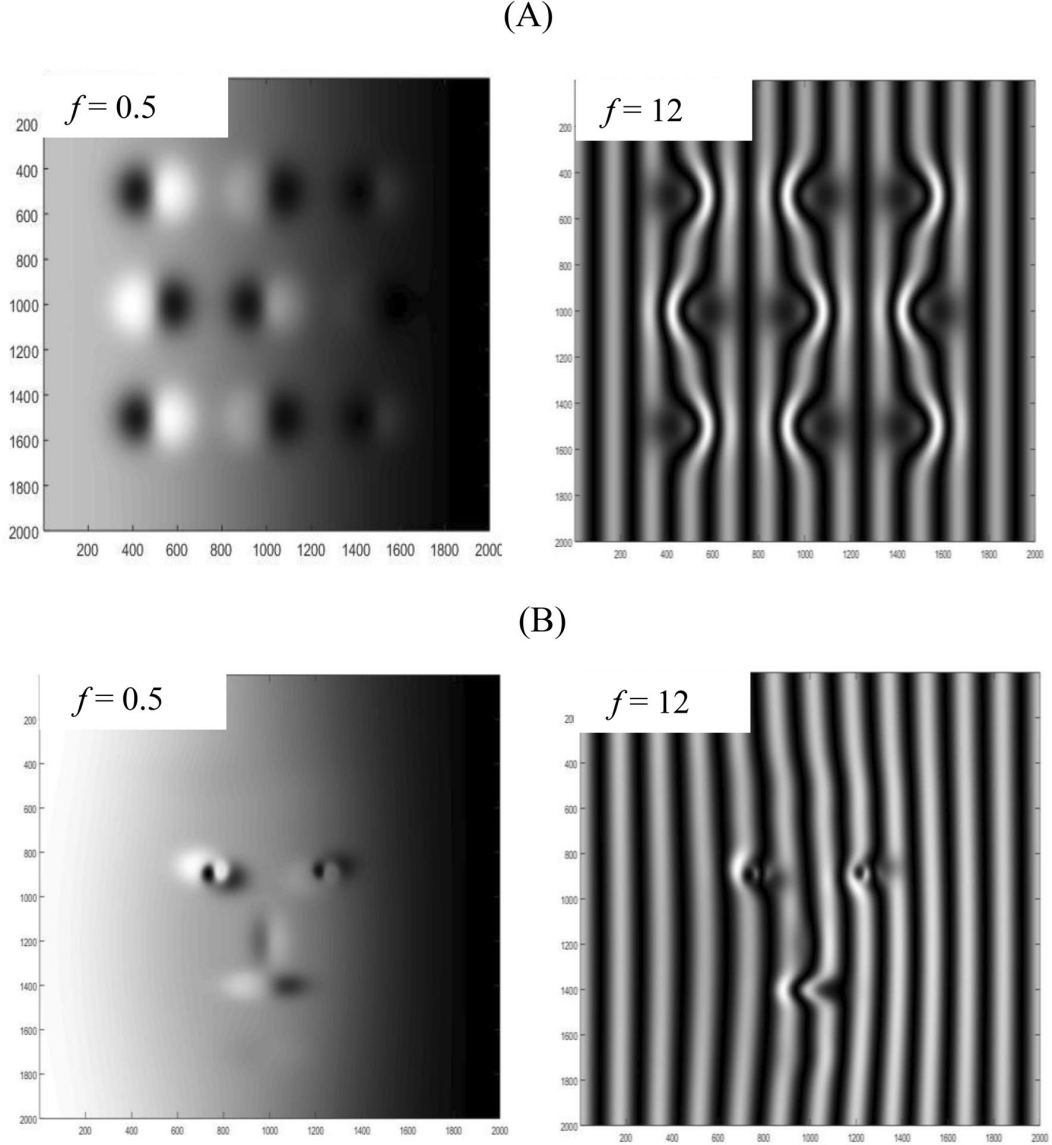
The projection of the interference pattern (Eq. (18)) on the two estimated objects (**A,B**) for *f* = 0.5 and 12.

Figure 4 illustrates the two estimated objects when they are projected with the intensity distribution given by Eq. (18) for fringes of (*f* = 0.5 and 12) and for the angle *θ* = 50°.

**Figure 5:**
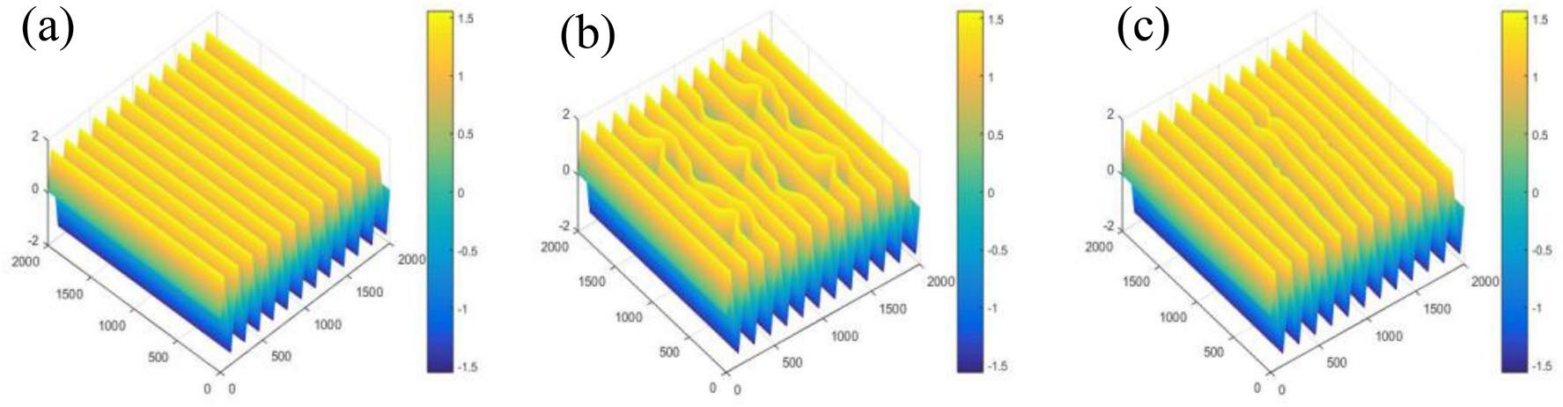
The wrapped phase maps of the (**a**) carrier fringes, (**b**) object (A) and (**c**) object (B) for the frequency *f* = 12.

Now, it is obvious that Eqs. (17)–(19) should be considered when dealing with Eqs. (6), (9)–(11) for recovering the object’s phase. For our case, Fig. 5 shows the obtained wrapped phase angle maps of the carrier and the two estimated objects (A, B) for *f* = 12 when we applied the above procedure.

**Figure 6:**
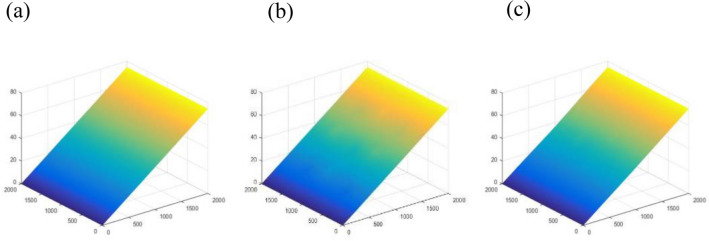
The unwrapped phase maps of the (**a**) carrier fringes, (**b**) object (A) and (**c**) object (B).

To obtain the unwrapped phase distribution of the maps shown in Fig. 5, Eqs. (6) and (10) are applied. Figure 6 shows the unwrapped phase distributions of the wrapped phase maps illustrated in Fig. 5.

**Figure 7:**
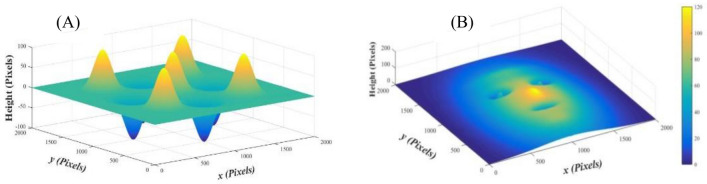
The two reconstructed objects (**A,B**).

By subtracting the unwrapped carrier’s phase distribution (i.e., Fig. 6a) from the unwrapped maps of the two objects according to Eq. (11), one can recover the phase objects *φ*(*x*,*y*). For obtaining the recovered objects with their heights in pixels, the object’s phase values are divided on (*ξ*), see Eq. (14). The two reconstructed objects are illustrated in Fig. 7.

## Limitations of the proposed method

### Summations in Eqs. (6) and (10)

**Figure 8:**
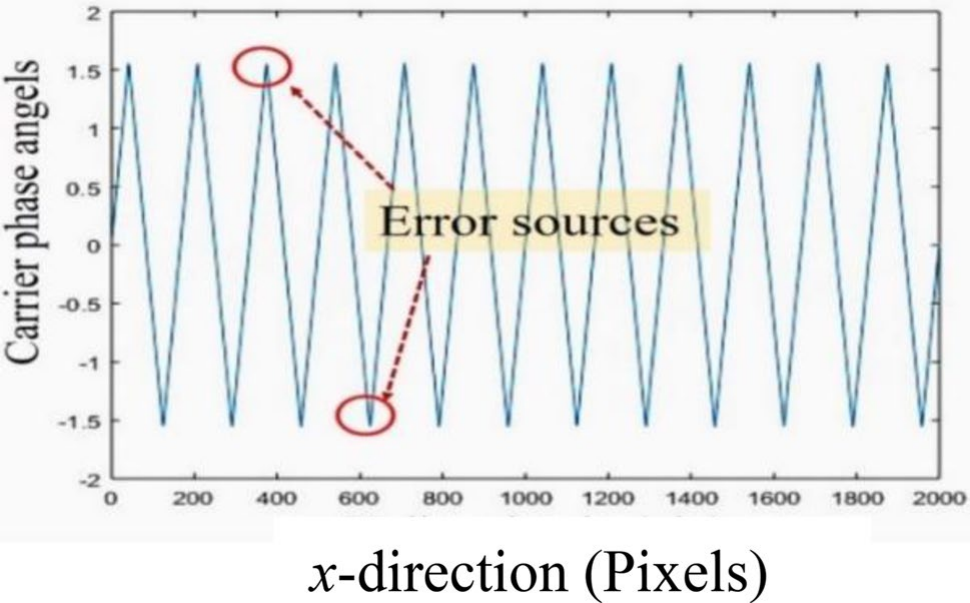
The sources of error due to the turning points when a phase angle tends to be − *π*/2 or *π*/2.

One can realize that when a phase angle tends to be − *π*/2 or *π*/2, there is an error due to these turning points. This error increases with increasing the number of the fringes (*f*) in the field while it can be minimized when, the number of pixels *M* in the field becomes higher, i.e., increasing the image’s resolution see Fig. 8. When we calculate the unwrapped resultant phase given by Eqs. (6) and (10), we can notice that it is probable that *δ*_*j*+1_ = *δ*_*j*_ or (*δ* + *φ*)_*j*+1_ = (*δ* + *φ*)_*j*_ which means that at the end of the summation, we will miss very small phase angles. The number of these missed phase angles depends on the number of the ± *π*/2 appeared on the phase angles and the value of the error each time is inversely proportional to the number of pixels (M). As demonstrated in Fig. 8, the error sources appear at phase angles of ± *π*/2.

**Figure 9:**
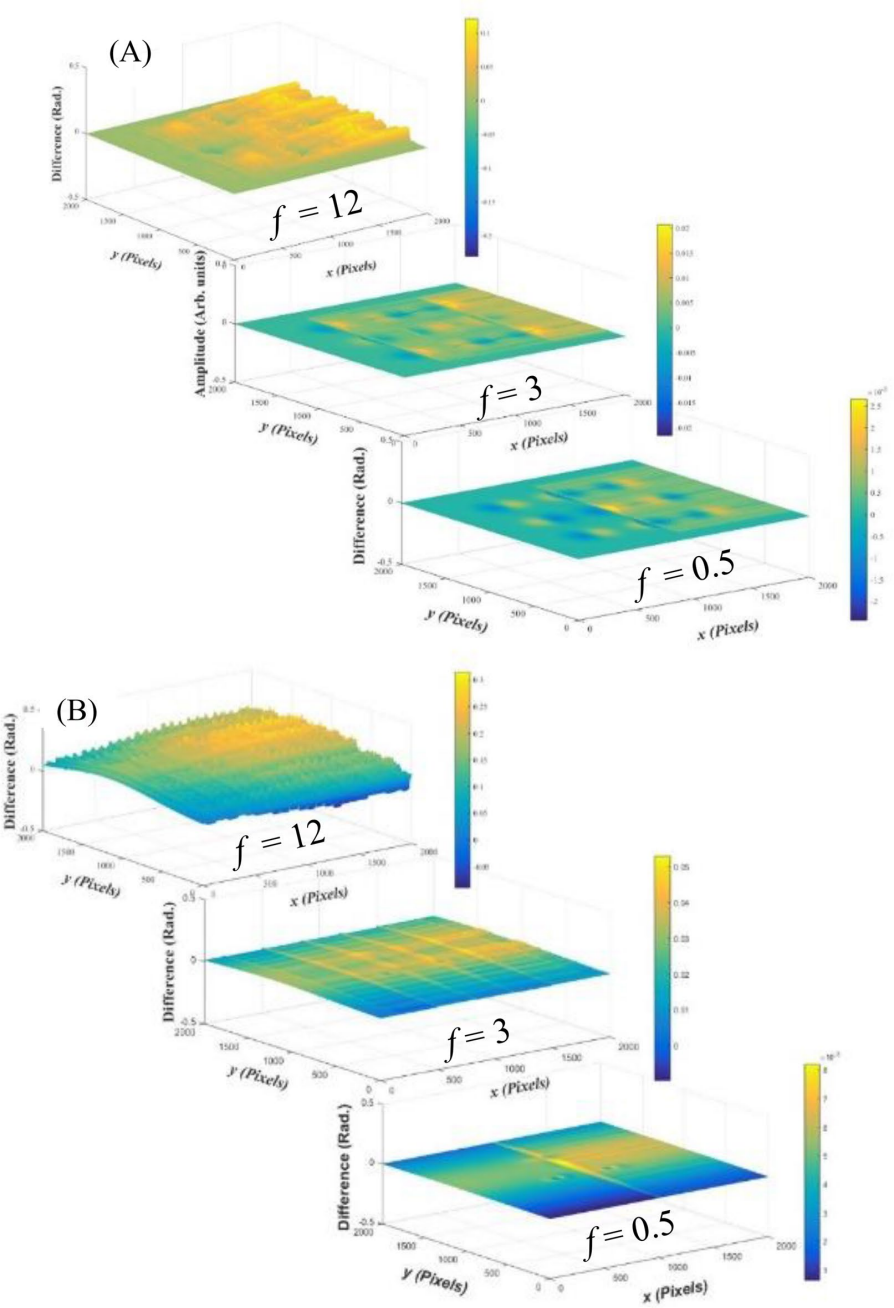
The differences between the simulated and the reconstructed objects for the two objects (**A,B**).

Therefore, we apply three estimated fringe patterns on each of the estimated objects. These patterns have different fringes numbers of (*f* = 0.5, 3 and 12). The corresponding shift values (*ϵ*) in the carrier phase are (1.57 × 10^–3^ Rad., 9.42 × 10^–3^ Rad. and 37.7 × 10^–3^ Rad.), respectively. The dimensions of each image are 2000 pixels × 2000 pixels. The reconstructed object is subtracted from the estimated one in each case to calculate the differences between the simulated and the reconstructed objects. These differences are illustrated in Fig. 9 for the two objects. As it is clear, the error is reduced from ± 0.1 Rad. (in case of *f* = 12) to ± 2.5 × 10^–3^ Rad. (in case of *f* = 0.5) for the object (A). For the object (B), the error is reduced from ± 0.3 Rad. (in case of *f* = 12) to ± 8 × 10^–3^ Rad. (in case of *f* = 0.5). This is an important result and recommends minimizing the number of the projected fringes (for a certain pattern’s width in pixels) in order to reduce the error in case of using the proposed approach.

### The boundaries of the incidence angle

The angle *β*, i.e., the incidence angle of the projected image given in Eq. (15), should be $\le \frac{\pi }{2}$ to make sure that there is no discontinuity in the recorded fringes. This means that the following relation should be always satisfied.

20 $$\frac{\pi }{2}-\left(\theta \pm \alpha \right)\le \frac{\pi }{2}.$$

Stating that the incidence angle (*θ*) should be always positive yields a limitation of *θ* to obey the following condition:

21 $$\theta \ge \pm \alpha .$$

When *α* is negative, the inequality (21) is always fulfilled. On the other hand, when *α* is positive, it must not exceed *θ*. Otherwise we get a discontinuity in the recorded fringes. In this case, we recommend increasing the value of the inclination angle (*θ*) of the projected light.

## Experimental verification of the proposed method

**Figure 10:**
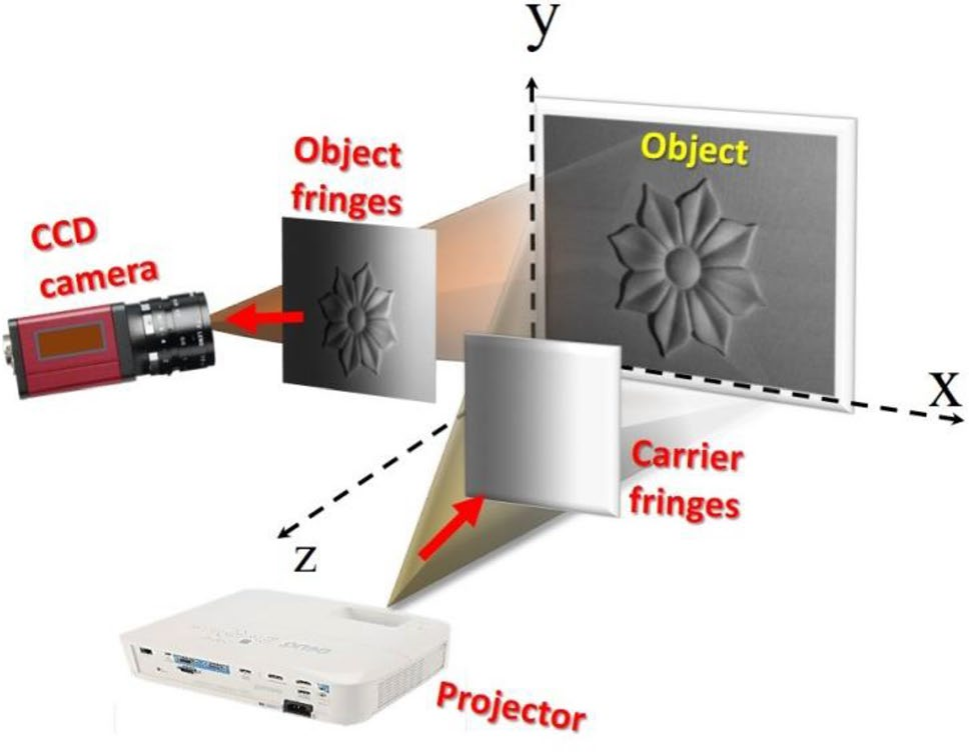
The experimental setup used to verify the proposed method.

**Figure 11:**
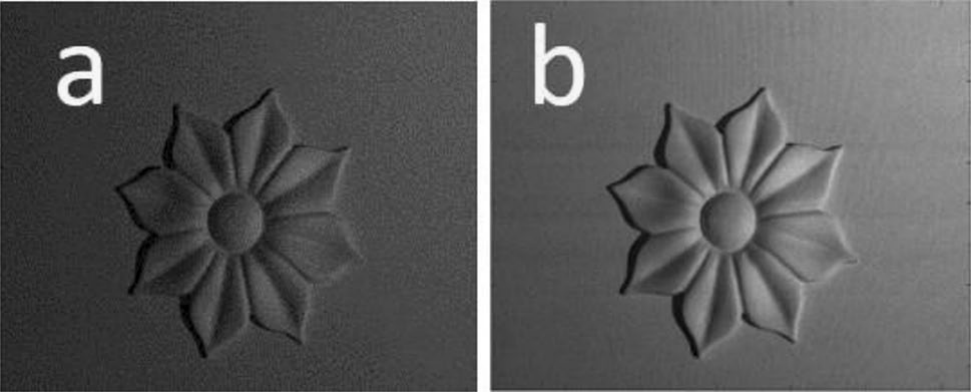
The (**a**) recorded and (**b**) digitally filtered images of the flower-like object when it is projected by a homogenous illumination.

In order to verify our proposed method, we prepared a fringe projection setup as shown in Fig. 10. A flower-like object is fixed on a flat background of dimensions ~ 39 cm × 29.5 cm. An image of homogeneous illumination is projected on the object by the aid of a “BenQ smart 1080p” projector. The incidence angle of the light on the object ranges from 41° to 50° with respect to the *x*-axis. The modulated image, by the object, is recorded using a “Marlin F145B2” CCD camera from Allied Vision® and having a pixel pitch 4.65 µm. As it was discussed in “Change of intensity due to the inclination of the incident light on the object” section, the prepared image of homogeneous intensity equals $2 \sqrt{{I}_{1 } {I}_{2}}$ is projected on the object. The recorded image in this case and its digitally filtered one are shown in Fig. 11a,b, respectively. The digital filtration process is applied for minimizing the noises appeared in the recorded images. One can notice that the homogeneous illumination is affected by the angle *β*(*x*,*y*), see Eq. (17).

**Figure 12:**
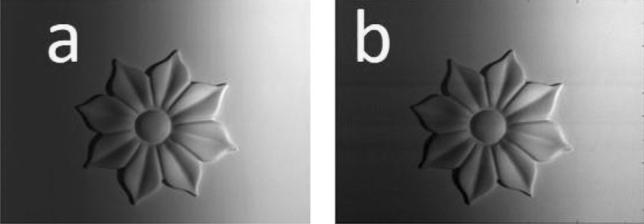
The (**a**) recorded and (**b**) digitally filtered images of the flower-like object when it is projected by the carrier fringes prepared according to Eq. (1).

**Figure 13:**
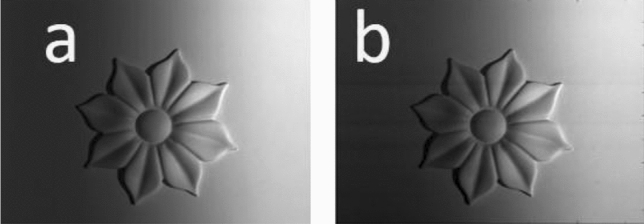
The (**a**) recorded and (**b**) digitally filtered images of the flower-like object when it is projected by the carrier fringes prepared according to Eq. (4).

Two interference patterns (carrier fringes) with a spatial frequency 0.5 and having $\epsilon$ = 0.1 Rad. are prepared using Eqs. (1) and (4). These patterns are projected on the object to be recorded by the CCD camera. The first modulated carrier fringes are recorded and shown in Fig. 12a while their digitally filtered ones are shown in Fig. 12b. Similarly, the $\epsilon$-shifted carrier fringes modulated by the object are shown in Fig. 13a while their digitally filtered ones are shown in Fig. 13b.

**Figure 14:**
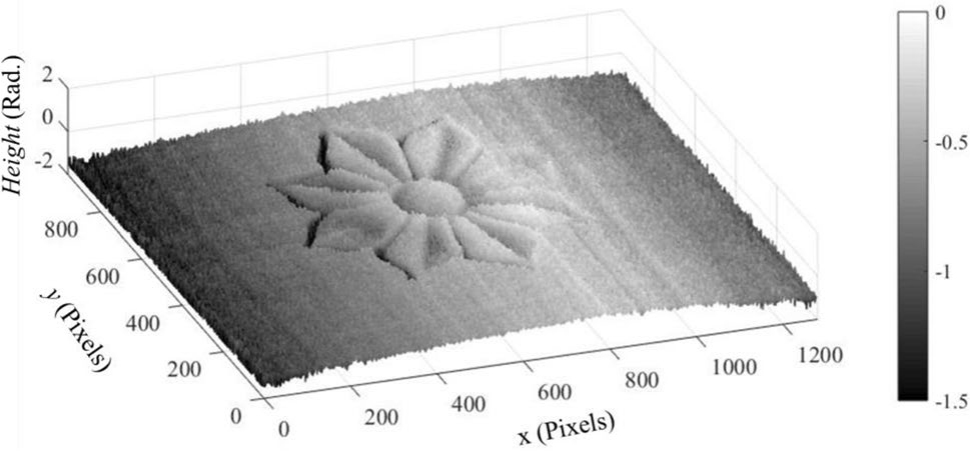
The resultant phase map $\varphi \left(x,y\right)+\delta \left(x\right)$ (in Rad.).

**Figure 15:**
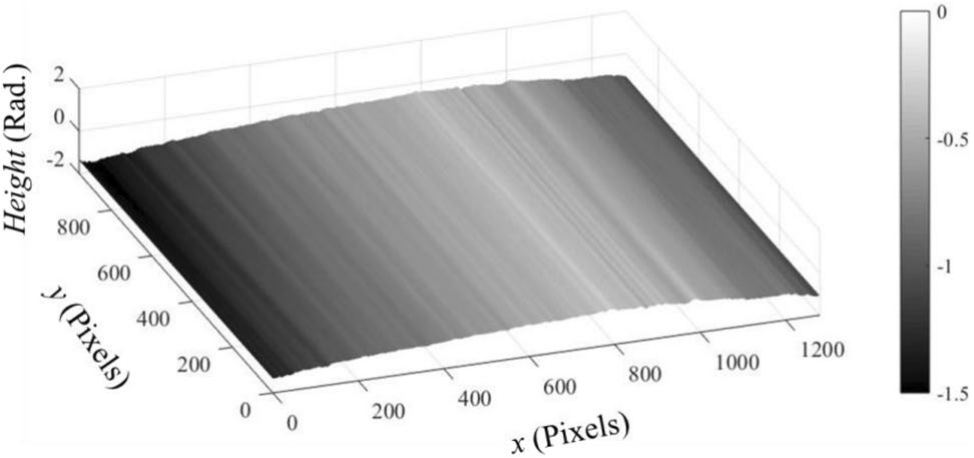
The carrier phase map $\delta \left(x\right)$ (in Rad.).

Now using Eq. (9), we can obtain the phase object $\varphi (x,y)$ in addition to the carrier phase $\delta \left(x\right)$ by substituting the values of both ${I}_{o}(x,y)$ and ${I}_{o,\epsilon }\left(x,y\right)$. We can see that the images in Figs. 12b and 13b represent the quantities ${I}_{o}(x,y)$ and ${I}_{o,\epsilon }\left(x,y\right)$, respectively. The value of the denominator $2 \epsilon \sqrt{{I}_{1 } {I}_{2}}$ is substituted from the image shown in Fig. 11b multiplied by the small phase shift $(\epsilon )$. The resultant phase map yielded from Eq. (9) is illustrated in Fig. 14. Similarly, the phase angle $\delta \left(x\right)$ of the background field, given by Eq. (5), are obtained, see Fig. 15.

**Figure 16:**
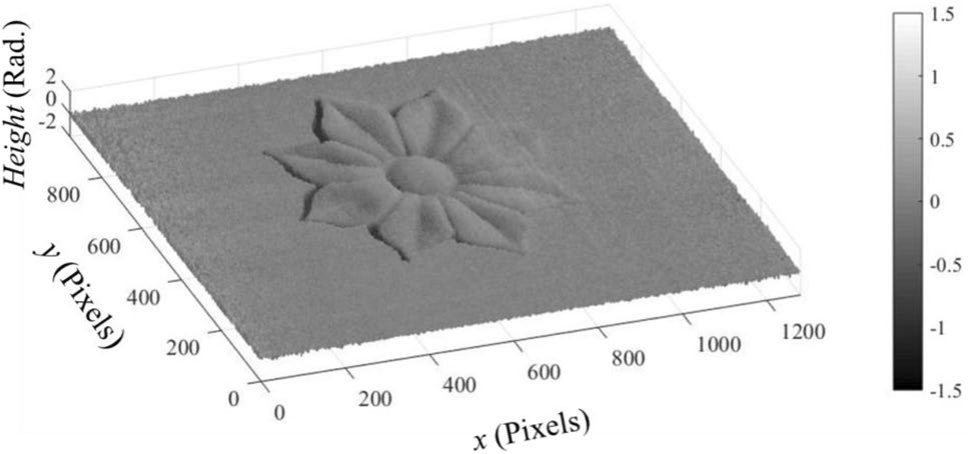
The object phase map $\varphi \left(x,y\right)$ (in Rad.).

It is obvious that the phase maps of the object and carrier don’t exceed one cycle. In this case, the object phase $\varphi (x,y)$ can be, directly, obtained by subtracting the phase map shown in Fig. 15 from that shown in Fig. 14. In this way, we obtained the $\varphi (x,y)$ as illustrated in Fig. 16.

**Figure 17:**
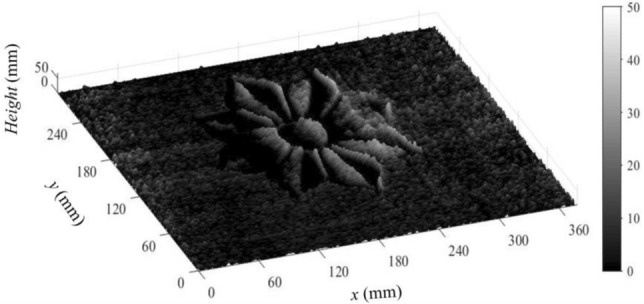
The object height (in mm) of the retrieved object.

Once we get the object phase map (in Rad.), we can use Eq. (14) to calibrate the phase values to obtain the object’s height (in mm), where:

22 $$Opject\, height \left(z\right)= \frac{\varphi \left(x,y\right) {\text{tan}}(\theta \left(x\right))}{\xi },$$

where, (ξ = 2πf/M (Rad./pixel)). Considering that *f* = 0.5 and M = 1280 pixels; ξ = 24.5 × 10^–4^ (Rad./pixel). Since the light coming from the projector is not parallel, $\theta \left(x\right)$ is found to be ranging from 41° (at the farthest projected point) to 50° (at the nearest projected point). This is taken into consideration when we calculated ${\text{tan}}(\theta (x))$. In this way and by considering the real metric dimensions, one pixel is found equivalent to 0.306 mm. Figure 17 shows the object height (in mm) of the retrieved object.

We have to conclude that the above experimental work is our first attempt to verify the presented theory. The amount of the problems we faced was a surprise for us, particularly, the accompanied noise which we could not exactly identify its source. This noise might be due to the sensitivity of the used camera and its fast response to the incident light. However, as a first trial, we believe that it is not so bad and this work demonstrates that the theory is valid and promising to be used with better instruments to give results that are more qualified.

## Conclusion

We presented an enhanced approach to recover the phase map of a three-dimensional object. The method is based on the differentiation of a single two-beam interference intensity distribution with respect to the phase angle. Only two estimated fringe patterns are projected on two estimated objects with an inclination angle 50°. A formula is proposed for unwrapping the phase values which are raised as inverse “sin”. This method shows that there is an error which grows with increasing the number of fringes in the projected pattern. This error is proved to be effectively minimized by reducing the frequency of the fringes in the projected field. The difference between the reconstructed and the estimated objects are determined. The difference in case of number of fringes in the projected pattern equals 0.5 doesn’t exceed 8 × 10^–3^ Rad. which is equivalent to *π*/375. The first attempt for experimental application, on a real 3D object, of the proposed approach is illustrated. By following the steps of the proposed method, we were able to retrieve the projected object and consequently, the tomography of the real object. The method presents a high sensitive way for recovering the phase map on a pixel scale. The obtained results are promising despite being having unexpected noise. Detecting the sources of the noise and solving this problem needs further work which is our outlook.

### Supplementary Information

Supplementary Information.

## Data Availability

All data generated or analyzed during this study are included in this published article and its Supplementary Information files.
