# Supplementary material for: Optical phase retrieving of a projected object by employing a differentiation of a single pattern of two-beam interference
Source: Sci Rep. 2023 Sep 8;13:14840. doi: 10.1038/s41598-023-41627-y (PMC10491839; doi:10.1038/s41598-023-41627-y)
Supplement: Supplementary file 1 — Supplementary Information. [file 41598_2023_41627_MOESM1_ESM.docx]

**Appendices for**

**“Optical phase retrieving of a projected object by employing a differentiation of a single pattern of two-beam interference”**

**Appendix (1)**

Based on equations (1) and (4), three pairs of interference patterns are simulated. The first pair is simulated with a frequency (*sf* = 0.5) while they are shifted by a phase shift 1.57×10^-3^ Rad. The second and third pairs have the spatial frequencies 3 and 12, respectively. The values of the phase shift are 9.42×10^-3^ Rad. and 37.7×10^-3^ Rad. for the second and third pairs of interference patterns, respectively.


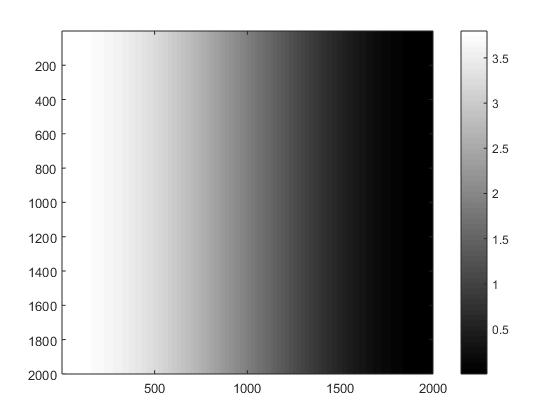

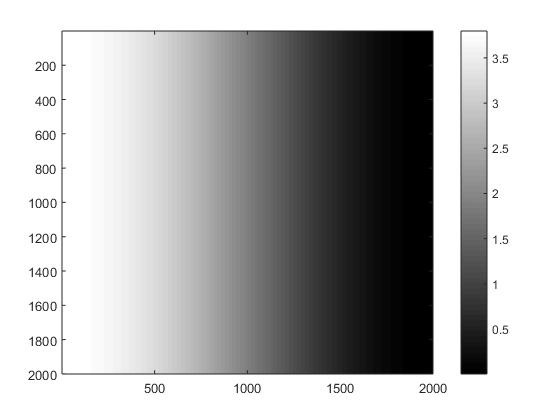


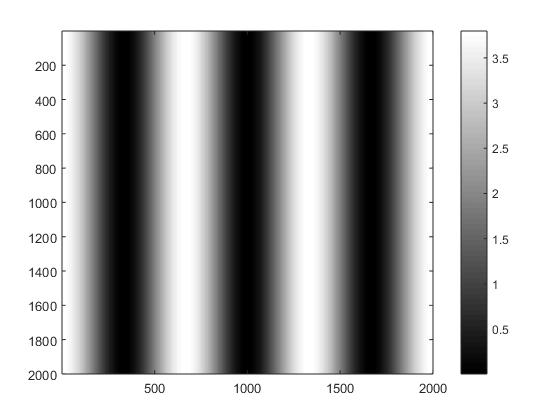

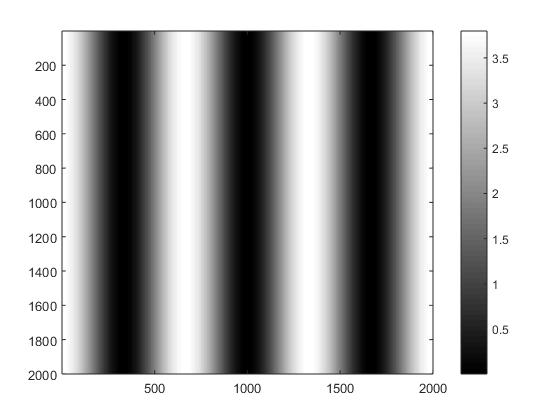


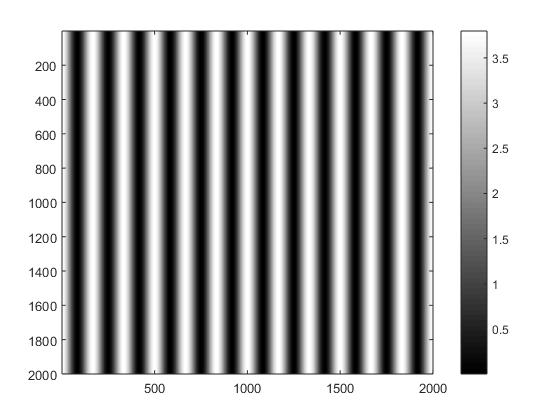

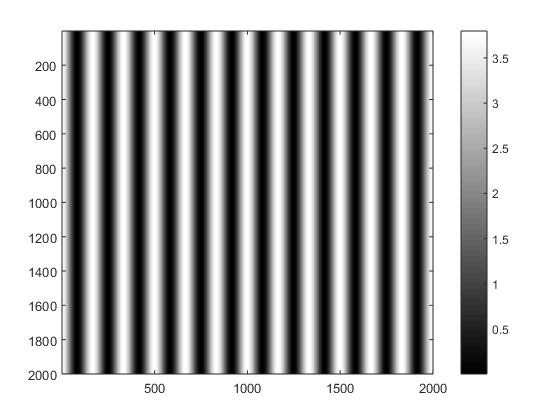


Supplementary figure (1): The estimated pairs of carrier fringes with different values of spatial frequency.

**Appendix (2)**

The following tables show the different parameters of the Gaussians used to estimate the two objects (A and B) based on equations (12) an (13). The next figures show the two estimated objects.

| **Estimated object (A)** | | | | | | |
| --- | --- | --- | --- | --- | --- | --- |
| *n* | *A_n_* | *ρ* | *a_n_* | *b_n_* | *w_xn_* | *w_yn_* |
| 1 | 100 | 0 | M/4 | M/4 | 85 | 85 |
| 2 | 100 | 0 | 2M/4 | M/4 | 85 | 85 |
| 3 | 100 | 0 | 3M/4 | M/4 | 85 | 85 |
| 4 | 100 | 0 | M/4 | M/2 | 85 | 85 |
| 5 | 100 | 0 | 2M/4 | M/2 | 85 | 85 |
| 6 | 100 | 0 | 3M/4 | M/2 | 85 | 85 |
| 7 | 100 | 0 | M/4 | 3M/4 | 85 | 85 |
| 8 | 100 | 0 | 2M/4 | 3M/4 | 85 | 85 |
| 9 | 100 | 0 | 3M/4 | 3M/4 | 85 | 85 |

Supplementary table (1): The different parameters of the Gaussians used to estimate the object (A).

| **Estimated object (B)** | | | | | | |
| --- | --- | --- | --- | --- | --- | --- |
| *n* | *A_n_* | *ρ* | *a_n_* | *b_n_* | *w_xn_* | *w_yn_* |
| 1 | 25 | 0 | M/2 | 3M/5 | 45 | 80 |
| 2 | 100 | 0 | M/2 | 3M/5 | 385 | 585 |
| 3 | -80 | -0.2 | 3.1M/5 | 4M/9 | 70 | 50 |
| 4 | -80 | 0.4 | 1.9 M /5 | 4 M /9 | 70 | 50 |
| 5 | -90 | 0 | M /2 | 2.8 M /4 | 85 | 40 |
| 6 | 75 | 0 | 3.1 M /5 | 4 M /9 | 30 | 30 |
| 7 | 75 | 0 | 1.9 M /5 | 4 M /9 | 30 | 30 |
| 8 | 12 | 0 | M /2 | 3.4 M /4 | 100 | 60 |
| 9 | 15 | 0.2 | M /2 | M /4 | 250 | 85 |

Supplementary table (2): The different parameters of the Gaussians used to estimate the object (B).

**(A)**


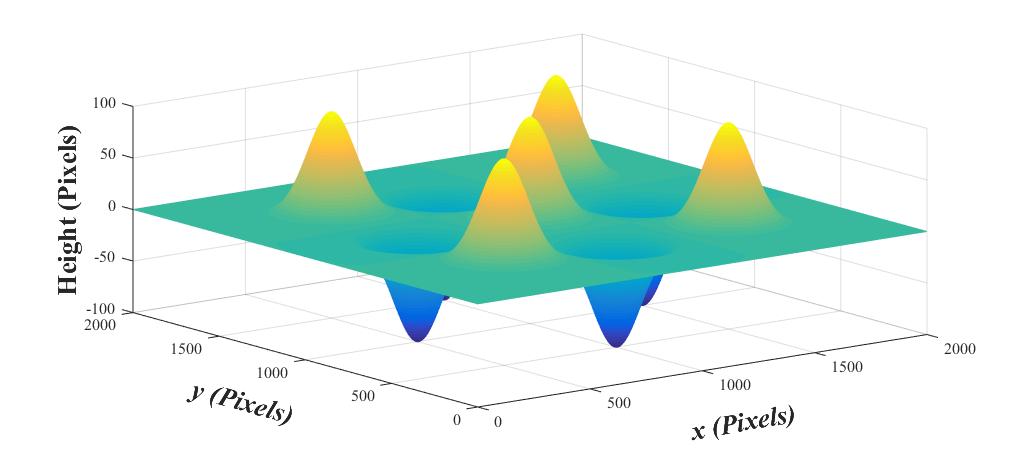


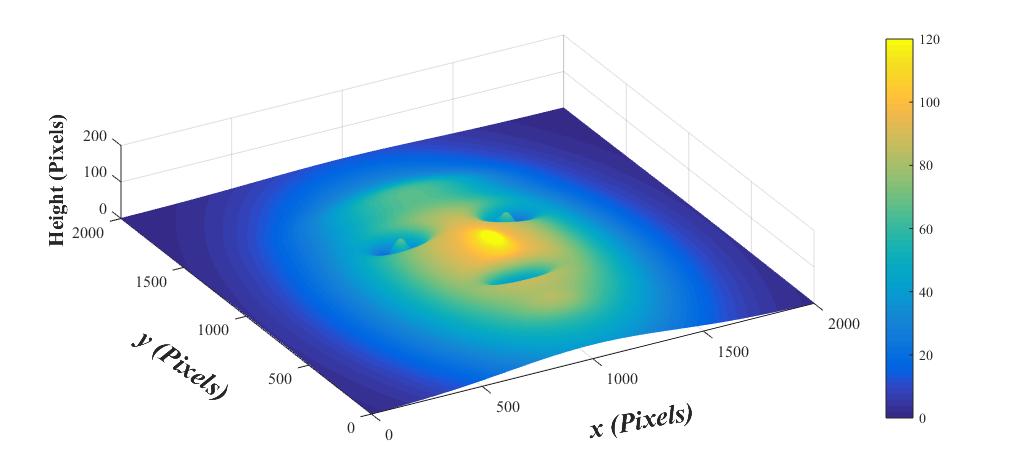


**(B)**

Supplementary figure (2): The estimated objects (A and B).
